# 

**Published:** 1896-05

**Authors:** 


**ESTABLISHED 1855.** **SUBSCRIPTION, $2.00.**

### ATLANTA MEDICAL AND SURGICAL JOURNAL.

VOLUME XIII. NEW SERIES. ATLANTA, GA., MAY, 1896. NO. 3.

